# The different ecological niches of enterotoxigenic *E* *scherichia coli*

**DOI:** 10.1111/1462-2920.13106

**Published:** 2015-12-21

**Authors:** Lucia Gonzales‐Siles, Åsa Sjöling

**Affiliations:** ^1^ Department of Infectious DiseasesInstitute of BiomedicineSahlgrenska AcademyUniversity of GothenburgGothenburgSweden; ^2^ Department of Microbiology, Tumor and Cell BiologyKarolinska InstitutetStockholmSweden

## Abstract

Enterotoxigenic *E* *scherichia coli* (ETEC) is a water and food‐borne pathogen that infects the small intestine of the human gut and causes diarrhoea. Enterotoxigenic *E*. *coli* adheres to the epithelium by means of colonization factors and secretes two enterotoxins, the heat labile toxin and/or the heat stable toxin that both deregulate ion channels and cause secretory diarrhoea. Enterotoxigenic *E*. *coli* as all *E*. *coli*, is a versatile organism able to survive and grow in different environments. During transmission and infection, ETEC is exposed to various environmental cues that have an impact on survivability and virulence. The ability to cope with exposure to different stressful habitats is probably shaping the pool of virulent ETEC strains that cause both endemic and epidemic infections. This review will focus on the ecology of ETEC in its different habitats and interactions with other organisms as well as abiotic factors.

## Introduction

Diarrhoea is the second leading cause of morbidity and mortality in children under 5 years old and is estimated to cause 2.5 billion cases and 760 000 deaths annually (Liu *et al*., 2012) Diarrhoeal disease is caused by bacteria, protozoa or viruses and can manifest as watery, secretory diarrhoea, bloody diarrhoea or persistent gastroenteritis. Episodes of diarrhoea have been shown to cause malnutrition and immune deficiencies, and severe and repeated episodes in newborns and toddlers can cause long‐term effects such as stunting and cognitive impairment (Niehaus *et al*., 2002). Hence, apart from the mortality rates, diarrhoea is affecting millions of lives globally and it remains to be a serious health problem.

Enterotoxigenic *Escherichia coli* (ETEC) is one of the most common causes of infantile and adult diarrhoea and traveller's diarrhoea in low‐income endemic countries and is estimated to cause 2.5 million cases and 700 000 deaths in children below 5 years of age (Qadri *et al*., 2005; Fischer Walker *et al*., 2010; Liu *et al*., 2012; Das *et al*., 2013; Kotloff *et al*., 2013; Lamberti *et al*., 2014). Enterotoxigenic *E. coli* diarrhoea is associated with poverty, lack of safe drinking water and inadequate sanitation (Curtis *et al*., 2000). In addition, ETEC may also cause outbreaks of food‐borne gastroenteritis (MacDonald *et al*., 2015). The hallmark of ETEC is the expression of one or two plasmid‐borne enterotoxins, the heat labile toxin (LT) and/or the heat stable toxin (ST) that both mediate deregulation of membrane ion channels in the epithelial membrane (Fleckenstein *et al*., 2010). Each ETEC isolate typically expresses one to three colonization factors (CFs) that mediate adhesion to the epithelium (Gaastra and Svennerholm, 1996). The most prevalent CFs are CFA/I and coli surface antigens 1–6 (CS1‐CS6), although CS7, CS14 and CS17 are also common (Gaastra and Svennerholm, 1996; Qadri *et al*., 2005; von Mentzer *et al*., 2014).

Enterotoxigenic *E. coli* diarrhoea is typically initiated through intake of contaminated food or drinks and is associated with low socioeconomic conditions and poor access to clean water and sanitation. The watery diarrhoea caused by ETEC can contain up to 10^9^ ETEC bacteria per ml (Å. Sjöling *et al*. unpubl. obs.), which poses a direct risk of transmission by shared toilets or through water transmission. Presence of ETEC in rivers, drinking water and in irrigation water has been shown (Begum *et al*., 2005; 2007; Lothigius *et al*., 2008; 2010; Ahmed *et al*., 2013), and ETEC is able to adhere firmly to fresh vegetables, which increases risk for transmission (Ethelberg *et al*., 2010; Shaw *et al*., 2011). Hence, ETEC is present in the environment and must adapt to, and survive, harsh conditions. On the other hand, the human gastrointestinal tract is an equally hostile environment and any successful pathogen needs to be able to utilize nutrients available in the gut and also to sense the environment for proper expression of virulence factors. Pathogenic bacteria infecting its host encounter a variety of host environmental factors that may influence virulence gene expression, e.g. fluctuations in pH, high osmolarity, bile, oxygen tension, different carbon sources, non‐digestible oligosaccharides, host immune factors such as antimicrobial peptides and the normal microbiota and its metabolites (Ferreyra *et al*., 2014). These factors are sensed by infecting bacteria and often regulate gene expression, morphology and physiology (Ferreyra *et al*., 2014). In addition, treatment with antibiotics might change the microbial population, and subinhibitory concentrations might even modulate virulence gene regulation in pathogens (Aminov, 2009). Enterotoxigenic *E. coli*, like all *E. coli*, are adaptable organisms able to survive and grow in different environments, and transmission through different ecological systems shapes survival mechanisms and virulence of ETEC strains. In this review, we will follow the transmission circle from water to ingestion, infection and back to water and discuss the different ecological niches that ETEC may encounter.

## ETEC ecology in environmental water

Enterotoxigenic *E. coli* is a food and water‐borne pathogen, typically ingested by contaminated food and drinking water, which has been shown repeatedly to be present in drinking and environmental water in endemic areas in Asia, Africa and Latin America (Begum *et al*., 2005; 2007; Lothigius *et al*., 2008; Patel *et al*., 2010; Nontongana *et al*., 2014). Faecal excretions from wildlife, livestock and human sources may contaminate irrigation water and soil and contribute to transfer of antibiotic resistance (Greig *et al*., 2015). When faecal bacteria are discharged into toilets or rivers and other water sources they are instantly exposed to colder and less nutritious environments and need to regulate their metabolism to cope with oligotrophic conditions. Entry into water immediately halts growth and division but bacteria can remain viable and persist for remarkable extended time periods, thus posing a serious threat for disease dissemination (Berthe *et al*., 2013; Jubair *et al*., 2012).

The ability to adapt to and persist in oligotrophic environments is inherent in *E. coli* (Na *et al*., 2006). Presence of *E. coli* in water is used worldwide as an indicator of potential faecal contamination of drinking water and distribution systems. However, *E. coli* is a diverse species divided into phylogroups A, B1, B2, D, E and the cryptic phylogroup C (Tenaillon *et al*., 2010). Recent studies suggest that specific resident environmental *E. coli* of phylogroup C are better adapted to survive in the environment (Luo *et al*., 2011). Other studies have shown that *E. coli* of phylogroup B1 persists longer in water than other groups (Berthe *et al*., 2013). We have recently shown that ETEC are mainly found in the A and B1 groups (von Mentzer *et al*., 2014). Enterotoxigenic *E. coli* is frequently isolated from environmental water samples (Begum *et al*., 2007; Lothigius *et al*., 2008; Akter *et al*., 2013). The ability to persist in water has been documented in ETEC, which can remain viable in both lake and seawater for months with retained gene expression (Lothigius *et al*., 2010). This suggests that water may be a both a reservoir for ETEC as well as a route of transmission. The frequent isolation of ETEC from water samples might provide a clue to why ETEC remains a common diarrhoeal pathogen globally.

Presence of coliforms, including ETEC, in different water bodies is dependent on chemical, physical and biological parameters such as available nutrients, temperature, pH and available ions. The frequency of *E. coli* contamination in water increases with environmental temperature (Black *et al*., 1982). Seasonal increase of ETEC in water corresponds to the warm rainy season (Lothigius *et al*., 2008; Ahmed *et al*., 2013). In Bangladesh, ETEC infections in children and adults have a typical biphasic pattern with epidemic peaks in the warmer periods in April and in August–September, before and after the monsoon rains. The incidence of ETEC infections seems to correspond to increased average temperature but not to occurrence of heavy rainfall (Black *et al*., 1982; Qadri *et al*., 2005). Enterotoxigenic *E. coli* has also been detected in seawater where areas of lower salinity in marine environments seem to favour occurrence of ETEC (Akter *et al*., 2013).

The water environment exposes bacteria to several stress cues, including cold shock, low ion concentrations and low osmolarity. Enterotoxigenic *E. coli* and other *E. coli* can deal with these stressful conditions by upregulation of genes involved in membrane stability, cold shock or survival and/or by adhering to abiotic or biotic surfaces to form biofilms (Costerton *et al*., 1999). In line with this *E. coli* was found to be 100‐fold more abundant in sediment than in water (Perkins *et al*., 2014), and the ecological niche for *E. coli*/ETEC in environmental water bodies is probably often in the form of biofilms that would provide survival advantages (Moreira *et al*., 2012). Biofilms are communities of bacteria attached to surfaces and surrounded by exopolysaccharides, proteins and nucleic acids (Costerton *et al*., 1999). Genes encoding biofilm extracellular matrix particularly poly‐beta‐1,6‐n‐acetyl‐D‐glucosaminecellulose (PGA), cellulose and colanic acid have been shown to be important for enterohaemorrhagic *E. coli* (EHEC) biofilm formation on sprouts and tomato roots grown in water (Matthysse *et al*., 2008). These genes, including curli, which is also implicated in biofilm formation, are expressed by certain ETEC and often induced by ambient temperatures, low ionic strength and nutrient limitation (Bokranz *et al*., 2005; Szabó *et al*., 2005). Hence PGA, cellulose, colonic acid and curli are expressed in conditions typically found in the environment (Beloin *et al*., 2008). Enterotoxigenic *E. coli* was found in biofilms formed in household drinking water sources in an endemic area during all months of the year (Ahmed *et al*., 2013). Higher incidences of ETEC‐positive biofilms were found during the peak period of the warm and rainy season when ETEC infections are more common, indicating that ETEC in biofilms are linked to the epidemic peaks (Ahmed *et al*., 2013).

However, it should also be mentioned that incubations in water microcosms have shown that ETEC and other bacterial pathogens survive in a planktonic state in water for months to years (Lothigius *et al*., 2010; Jubair *et al*., 2012). Enterotoxigenic *E. coli* apparently persist in sterile filtered lake water without forming biofilms (Lothigius *et al*., 2010). The number of bacteria detected by polymerase chain reaction methods remains stable for prolonged periods in sterile microcosms although culturable bacteria decline at a faster pace (Lothigius *et al*., 2010; Mauro *et al*., 2013). A small fraction of the remaining bacteria may enter a persister state of resting cells (Lewis, 2005). The bacterial persisters were first described as small subpopulations in bacterial cultures that display increased resistance to antibiotics. Persisters are a state of dormant cells that can be isolated in low frequency from exponential‐phase cultures, and in up to 1% of cells in bacterial biofilms and stationary‐phase cultures. Persister‐like bacterial cells form in water and might thus provide long‐term transmission properties of surviving pathogens (Jubair *et al*., 2012). We have observed that the *csrA* (carbon storage regulator) transcripts increase over time when ETEC are incubated in water (Å. Sjöling *et al*. unpubl. obs.). CsrA controls a large variety of physiological processes such as central carbon metabolism, motility and biofilm formation and has been linked to survival (Romeo, 1998; Beloin *et al*., 2008). One can speculate that the water environment gradually shapes bacteria with increasing capability to survive the nutrient poor environment. The ability to adapt and survive water transmission is therefore important for successful spreading of water‐borne pathogens.

## The surface of leaves and fruit provide a specialized ecological niche

Transmission of ETEC is specifically known to be associated with contaminated weaning foods among younger children in developing countries, in a study in the early 1980s, authors found that over 40% of food prepared for children contained *E. coli*, and a correlation between intake of contaminated food and incidences of diarrhoea was found (Black *et al*., 1982). Food‐borne transmission of ETEC is probably also often mediated through fresh produce irrigated by contaminated water. Enterotoxigenic *E. coli* diarrhoea has emerged as a diarrhoeal pathogen causing outbreaks of gastroenteritis after intake of salads and buffets in industrialized countries (MacDonald *et al*., 2015). Rinsing may not be sufficient to remove adherent bacteria since pathogenic *E. coli*, including ETEC, adheres firmly to lettuce and leafy vegetables. In ETEC, this adhesion occurs by means of the flagella (Shaw *et al*., 2011), while other pathogenic *E. coli* use adhesion factors (Berger *et al*., 2009).

Aquatic plants including algae may serve as reservoirs of ETEC in natural water resources (Singh *et al*., 2010; Quero *et al*., 2015). The numbers of ETEC in surface water was observed to vary significantly among different sampling points, but higher numbers of bacteria were found to be associated with plants than in water (Singh *et al*., 2010). Occurrence of ETEC in aquatic flora supports the view that aquatic plants could serve as secondary habitat for water‐borne bacteria due to the availability of nutrients from the decomposing tissue (Singh *et al*., 2010). Exposure to lysates of lettuce‐induced expression of *E. coli* genes involved in uptake of maltose, galactitol, glucose and galactose indicating that plants are utilized as carbon source (Kyle *et al*., 2010). However, exposure to intact lettuce leaves did not induce this effect, but rather upregulated the *gadABC* operon involved in acid resistance and the stress induced chaperon *HdeA* (Fink *et al*., 2012). Interestingly, both these genes were found to be upregulated in ETEC exposed to acidic pH, a condition that favours accumulation of ETEC LT in the periplasm but totally inhibits toxin secretion (Gonzales *et al*., 2013). It is possible that ETEC adhering to fresh produce encounter an environment that prepares them to be infectious and to store preformed LT. The ecological niche of plant leaves (the phylloplane) has a pH that might vary between plant species. Thus, the effects of bacterial adhesion to various plants with different surface pH and its effect on virulence expression would be intriguing to investigate.

## Transmission through the acidic gastric and alkaline duodenum shapes toxin expression and secretion in ETEC

Ingestion of ETEC through food and drinking water expose the bacteria to the host environment characterized by acidic exposure in the stomach followed by bile and bicarbonate influx in the duodenum and an increasingly anaerobic environment. The most influential change is probably the exposure to body temperature and virulence factors of pathogenic bacteria are typically induced in response to 37°C.

The transport through the gastrointestinal tract first exposes ETEC to low pH in the gastric lumen. In order to infect successfully human hosts, all pathogens need to adapt to low pH conditions by expression of genes that confer protection against acidic conditions (Foster, 2004). *Escherichia coli* in general have four systems that provide resistance against acidic conditions (Foster, 2004; Lund *et al*., 2014). It has been reported that certain pathogenic *E. coli*, i.e EHEC O157:H7, have increased resistance towards acid compared with other pathovars and commensal *E. coli* (Arnold and Kaspar, 1995); however, *E. coli* in general is able to survive the extreme acidic conditions present in the gastrointestinal (Lund *et al*., 2014).

Transport to the duodenum and small intestine instead exposes infecting pathogens to an alkaline flush through bile and bicarbonate (Begley *et al*., 2005). A large number of adaptive strategies have been developed for alkaline pH homeostasis including increased metabolic acid production through amino acid deaminases, and sugar fermentation to reduce the cytosolic pH (Padan *et al*., 2005). Other factors include increased adenosine triphosphate (ATP) synthase expression that couples H + entry to ATP generation to maintain protons within the cytosol, changes in membrane properties and increased expression and activity of monovalent cation/proton antiporters (Padan *et al*., 2005). Among these strategies, monovalent cation/proton antiporters play an essential and dominant role in alkaline pH homeostasis in many bacteria (Padan *et al*., 2005).

Under acidic and alkaline stress conditions, bacteria must maintain a cytoplasmic pH range of pH 7.2–7.8, compatible with optimal function of the cytoplasmic proteins (Zilberstein *et al*., 1984). To maintain pH homeostasis, bacteria are able to acidify or alkalinize the cytoplasm relative to the external milieu (Padan *et al*., 2005; Lund *et al*., 2014). Studies on the proteome profile of ETEC and *E. coli* K‐12 under neutral and alkaline conditions showed upregulation of proteins belonging to the electron transport chain that are involved in the maintenance of a stable internal pH (L. Gonzales‐Siles *et al*., submitted; Maurer *et al*., 2005). Additionally, upregulation of the DsbA protein and the Sec‐dependent transport system was found under alkaline conditions (L. Gonzales‐Siles *et al*., submitted). DsbA is involved in the assembly of the mature LTAB_5_ holotoxin (Mudrak and Kuehn, 2010). The LTA and LTB peptides are transported through the inner membrane to the periplasm via Sec‐dependent transport. External pH has been shown to have an effect on both the production and secretion of ETEC LT (Gonzales *et al*., 2013). Under acidic conditions, secretion over the outer membrane is inhibited while it increases significantly at neutral and basic pH conditions (Gonzales *et al*., 2013). Thus, improved and/or increased assembly and transport of LT could explain the reported elevated levels of secretion of LT at high pH.

The general view on LT secretion is that ETEC strains are not proficient at secreting LT into the extracellular media (Clements *et al*., 1985), and ETEC are instead suggested to keep the majority of the toxin produced attached to the membranes or in the periplasm (Wensink *et al*., 1978; Kunkel and Robertson, 1979). Our recent results instead indicated that ETEC is able secrete up to 60% of LT under alkaline conditions (Gonzales *et al*., 2013). In fact, highly alkaline surface microclimates close to the epithelium in the small intestine have been described (Mizumori *et al*., 2009). It is possible that ETEC use the pH gradient in the gastrointestinal tract to modulate its toxin production. The LT production and bacterial growth is increased in response to the encounter of the highly acidic stomach environment, but the toxin is not excreted from the periplasm until ETEC reaches its proper alkaline niche of infection close to the epithelium in the small intestine (Gonzales *et al*., 2013).

## The gastrointestinal tract provide ecological niches with microenvironments that induce virulence in ETEC

Enterotoxigenic *E. coli* causes an infection in the small intestine where it adheres to the epithelium by means of colonization factors and subsequently secretes the two toxins LT and ST that cause secretory diarrhoea through induction of efflux of water and electrolytes (Qadri *et al*., 2005). Stable toxin and LT bind to the enteric receptors guanylate cyclase and GM1, respectively, which are situated in the epithelial apical membrane. The binding of ST and LT to these receptors leads to the increase of intracellular cyclic guanosine monophosphate (cGMP) and cyclic adenosine monophosphate (cAMP) levels respectively (Fleckenstein *et al*., 2010). In strains that express both LT and ST, a synergistic effect has been observed on fluid secretion in the intestine (Read *et al*., 2014).

Adherence is linked to disease and provides close contact to the gut epithelium. Adherence mechanisms and/or biofilm production of ETEC isolates have been shown to be associated with colonization factors, and multiple CF genes located on the plasmid as well as the chromosome have been implicated to participate in primarily adhesion but also recently in biofilm development. The ETEC CFs are not expressed at ambient temperatures but induced at 37°C (Gaastra and Svennerholm, 1996). Enterotoxigenic *E. coli* strains expressing CFA/I, CS1, CS2 or CS3 was reported to produce higher levels of biofilm due to higher hydrophobicity than its corresponding mutant strains (Liaqat and Sakellaris, 2012). Other studies have suggested that the TibA adhesin, in addition to its ability to promote bacterial binding to and invasion of human cells, possesses alternative virulence properties including biofilm formation (Sherlock *et al*., 2005). Since CFA/I, CS1‐CS3, TibA and CS21, which are also implicated in ETEC biofilms, are only produced by certain ETEC clones, it is possible that ETEC infections differ from each other with respect to ability to form biofilms on the gut epithelium. However, the clinical significance of biofilm formation during infection has not been thoroughly studied in ETEC.

## Osmolarity and physiological temperature

The gut environment provides several cues that induce virulence expression in ETEC. Interestingly, LT and ST toxins are differently regulated by some of these host environmental factors while other factors have similar effects. Toxins and colonization factors are inhibited by the H‐NS nucleoid‐associated protein, a global repressor central in regulation to physiological processes of bacterial cells including changes in temperature, osmolarity, desiccation and pH (Schröder and Wagner, 2002). H‐NS is often a major repressor of virulence factors in bacteria and relieves its repression when bacteria enter the physiological temperature and higher osmolarity present in the gut. H‐NS is known to inhibit *eltAB* expression by binding to a downstream regulatory element at low temperatures and low osmolarity (Trachman and Yasmin, 2004). H‐NS also directly represses the expression of *estA* genes encoding the two ST variants STp and STh, and this repression can be relieved, in an H‐NS dependent manner, by increased osmolarity such as addition of salt (Haycocks *et al*., 2015). The same type of regulation is found in operons encoding ETEC colonization factors, e.g CFA/I (Sanchez and Holmgren, 2005).

## Glucose

The preferred carbon source glucose is present in the upper parts of the small intestine but gradually decline further down due to active uptake of glucose through glucose transporters present on the epithelial cells. Presence of glucose suppresses synthesis of cAMP in prokaryotes (Bruckner and Titgemeyer, 2002). Cyclic AMP is second messenger that bind to and activate the cAMP receptor protein (CRP)–cAMP complex formation. Cyclic AMP receptor protein is a major transcription factor in *E. coli* and is involved in regulation of over 200 genes (Lee and Busby, 2012), it is also typically involved in regulation of virulence factors in a number of enteric pathogens. Interestingly, CRP‐mediated regulation is different for the two ETEC toxins ST and LT. Cyclic AMP receptor protein directly activates transcription of *estA* genes by binding to the promoter, but CRP represses *eltAB* transcription. In addition, although we, and others, have shown that CRP is involved in repression of *eltAB* using crp mutant strains (Bodero and Munson, 2009; Gonzales *et al*., 2013), a recent study showed that this repression is indirect and not dependent on the crp binding sites upstream of the *eltAB* promoter (Haycocks *et al*., 2015). The effects of CRP and glucose are often inverted, so glucose in the environment induces *eltAB* expression and inhibits expression of *estA* (Haycocks *et al*., 2015; Sahl *et al*., 2015). Glucose at a physiological and optimal concentration for LT production (0.25%) has also been reported to enhance bacterial adherence to epithelial cells through the promotion of LT production indicating that LT also facilitate adherence (Wijemanne and Moxley, 2014). Hence, varying glucose levels in the gut could influence toxin gene expression and possibly severity of disease.

## Bile

Bile and its specific bile components is another inducer of virulence factors in ETEC (Nicklasson *et al*., 2012). Bile is secreted into the small intestine from the gall bladder as a complex mixture of electrolytes, bile salts, phospholipids, cholesterol and bilirubin (Gunn, 2000; Begley *et al*., 2005). Bile salts themselves are a mixture of primary acids such as taurocholic, glycocholic, chenodeoxycholic and cholic acids in conjunction with sodium, and their concentration varies between 0.2–2% in the small intestine (Gunn, 2000). Bile in high concentrations is bactericidal, and a number of genes are involved in bacterial resistance to bile (Begley *et al*., 2005). However infecting enteropathogens may use bile as a signal that they have reached the human small intestine. Crude bile extracts may differ in their composition since the concentration of individual bile salts *in vivo* is affected by the nutritional status of the host, e.g. the ingestion of a fatty meal will increase concentration of bile in general, and a protein‐rich diet will increase the ratio of tauro‐conjugated bile acids and decrease the ratio of gluco‐conjugated bile acids (Begley *et al*., 2005). The concentration of bile in the intestinal lumen is also generally lower in malnourished patients (Begley *et al*., 2005). Bile salts can induce the expression of virulence factors in ETEC (Sjöling *et al*., 2007). The bile acid glycocholate hydrate and sodium deoxycholate have been shown to induce dose‐dependent expression of the ETEC colonization factor CS5 (Nicklasson *et al*., 2012). Enterotoxigenic *E. coli* CF expressions vary in response to bile, some are also induced by bile components while others are not affected at all by bile (Sjöling *et al*., 2007). Glycocholate hydrate would be expected to increase in individuals with carbohydrate rich diets often found in poor populations (Begley *et al*., 2005). Hence bile induced transcription might be important to study in relation to global health, since the diet might influence the severity of disease during infection of enteropathogens that regulate virulence in response to bile. Upregulation of *estA* encoding ST toxin was reported in the presence of bile in ETEC (Sahl and Rasko, 2012). We have observed that secretion of ST is induced by amino acids and bile salts, although the effect of bile differs on different types of ST toxins (E Joffre *et al*., submitted, L Gonzales‐Siles and Å. Sjöling, unpubl. data). Interestingly, ETEC expressing STp in combination with CS6, are significantly associated to diarrhoea in adults, and STp is induced in the presence of bile (E Joffré *et al*, submitted, Isidean *et al*., 2011). These data suggest that ETEC virulence depend both on bile compositions in the individual gut, but also on the type of ETEC strain causing the infection and its ability to respond to bile components.

## Carbon sources and mucins

Although glucose has been studied extensively, there are indications that pathogens also respond to the presence or absence of other carbon and energy sources. *Escherichia coli* which colonize the intestine of mammals have a metabolism adapted to use mucus as a nutrient and are able to grow on several mucus‐derived sugars and oligosaccharides (Chang *et al*., 2004). For instance, genes involved in glycogen, maltose and maltodextrin catabolism were found to be significantly upregulated in *E. coli* during growth on mucus, a condition that mimics nutrient availability in the intestine (Jones *et al*., 2008).

*Escherichia coli* and ETEC strains generally express the gene *yghJ* also known as *sslE*, which encodes a mucinase located upstream of the type II secretion system (T2SS), which is involved in the secretion of LT toxin (Sjoling *et al*., 2015; Valeri *et al*., 2015). YghJ/SslE is a highly conserved metalloprotease that influences intestinal colonization of ETEC by degrading the major mucins in the small intestine, MUC2 and MUC3 (Luo *et al*., 2014). By degrading the protection of the mucus layer YghJ/SslE allows efficient access to small intestinal enterocytes and thereby optimize adhesion to the epithelium and delivery of toxins.

Mucins also contain oligosaccharide moieties. There is increasing evidence that oligosaccharides and glyco‐conjugates present in breast milk and on mucins might affect virulence‐related abilities of pathogenic microorganisms (Pacheco and Sperandio, 2015). Mucin‐derived oligosaccharides like lactulose, raffinose and fucose that can be used by certain ETEC strains, are cleaved off from mucin and utilized by commensal bacteria such as *Lactobacillus, Bifidobacterium* and *Bacteroides* that may compete with infecting pathogens for space and nutrients (Pacheco and Sperandio, 2015; Conway and Cohen, 2015; Å Sjöling *et al*., unpubl. obs.). Is has also been suggested that oligosaccharides have structures that resemble the binding sites of pathogenic bacteria and hence may hinder adherence and subsequent disease (Kunz *et al*., 2000).

## The microflora may modulate establishment of infection and recovery

The human gastrointestinal tract harbours a diverse microbial community, which constitutes the microbiota. The microbiota is important for metabolic functions such as production of vitamins, metabolites and short chain fatty acids (den Besten *et al*., 2013). It shapes the development of the immune system in newborns and it protects against, and competes with, pathogenic species. Pathogens in the gastrointestinal tract have to interact with the normal microbiota and in addition there is a growing awareness that pathogens like ETEC also have to interact with other diarrhoeal pathogens since co‐infections are detected repeatedly. Recently, a number of studies have focused on determination of the microbiome as well as the presence and/or relative abundance of pathogens in diarrhoeal stool compared with healthy individuals (Kotloff *et al*., 2013; Taniuchi *et al*., 2013; Pop *et al*., 2014). The results revealed a more complex situation than previously anticipated and have also enlightened the need for quantitative analyses of commensal and pathogen load in order to determine aetiology. The microbiota has been shown to shift to lower diversity and a predominance of specific genera in the diarrhoeal gut (Monira *et al*., 2013; Pop *et al*., 2014). A study by Taniuchi and colleagues (2013) revealed that both symptomatic and healthy children carry multiple pathogens during their first year of life in Bangladesh. Monira and colleagues (2013) showed how cholera infection shifts the microbiota resulting in e.g less *Bifidobacteria*. The genera *Bifidobacterium* and *Lactobacillus* have been used as probiotics and have been implicated as protective against gastrointestinal pathogens. *Lactobacilli* were found to protect against Shigellosis (Lindsay *et al*., 2015). *In vitro* assays also indicated that *Lactobacilli* inhibits ETEC (Tsai *et al*., 2008). Diarrhoeal episodes thus seem to deplete some genera that are considered important for a healthy gut.

Diarrhoea affects the poor and malnourished population in low‐income countries and poses a particular risk of morbidity and mortality for children. Healthy Bangladeshi children had a higher diversity of gut microbiota than malnourished children (Monira *et al*., 2011). The phylum *Proteobacteria* increased in malnourished children while *Bacteroidetes* decreased. Particularly the *Proteobacteria* genera *Escherichia* and *Klebsiella* increased in the malnourished children although they did not display signs of diarrhoea. A recent study observed reproducible changes in the microbial community structure after ETEC and cholera infection. The authors found that diarrhoeal infectious species more or less could dominate the microbiota early in the infection. Re‐colonization of facultative anaerobic bacteria that metabolize oxygen introduced to the intestinal system through the diarrhoea then has to occur before the normal commensal obligate anaerobes can begin re‐colonizing the gut. Interestingly, a transient peak of *Bacteroides* was observed when the patients started to recover 7–10 days after acute infection and the microbiota was restored to normal diversity and structure after 1 month (David *et al*., 2015). It is clear that future studies of the microbiota in relation to diarrhoeal diseases will offer novel insights into the complex ecological niches that ETEC and other pathogens encounters in the gut during acute diarrhoeal disease and at recovery.

In addition, the interaction of several pathogens present might either enhance or decrease disease. Co‐infections of rotavirus, *Giardia lamblia* and ETEC were found to increase symptoms in children in Ecuador (Bhavnani *et al*., 2012). However, presence of *Giardia* is contradictory, its immunomodulating properties have been linked to protection although epidemiological studies have clearly defined it as a pathogen (Solymani‐Mohammadi and Singer, 2010). Indeed studies have detected *Giardia* in higher frequencies in asymptomatic children (Kotloff *et al*., 2013). In ETEC‐induced diarrhoea, the *E. coli* population could be dominated by a single clone of ETEC in some individuals, while co‐infections of several ETEC clones were observed in other patients (Sahl *et al*., 2015). It is possible that interactions between different ETEC clones occur under such co‐infections, which poses an interesting new aspect to ETEC infections.

## Nutrient status and immunomodulating additives may have impact on ETEC infection – lessons learned from porcine ETEC infections

Enterotoxigenic *E. coli* causing disease in humans have a tropism for the human intestine through the expression of colonization factors specific for human receptors. Enterotoxigenic *E. coli* is however also a zoonotic disease affecting piglets and calves, and most of the studies on ETEC gut ecology has been performed in piglets due to the economic impact of diarrhoeal deaths in weaning piglets. Porcine ETEC is responsible for neonatal and post‐weaning diarrhoeal infections (Berberov *et al*., 2004). In animals, supplementation of various nutrients, trace elements and vitamins have been evaluated for improvement of host defense or reduced diarrhoea including, e.g supplementation of amino acids (Tang *et al*., 2014), black tea extracts (Bruins *et al*., 2011), zink (Shen *et al*., 2014) and other additives. Although food additives have been less investigated in humans, it might be interesting lessons to learn from the porcine variants of ETEC since they share some toxin variants.

## Concluding remarks and prospective

**Figure 1:**
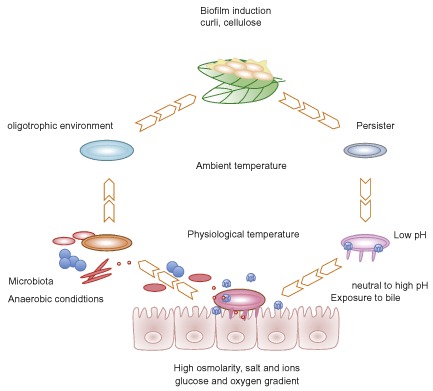
The ecological niches of ETEC. Water and food transmission exposes ETEC to ambient temperatures and nutrient poor environments with low osmolarity. The conditions favour induction of biofilms and survival factors and ETEC usually ceases growth and division. Entry into the gastrointestinal tract exposes ETEC to gradients of pH, osmolarity, nutrient concentrations, bile salts and an increasingly anaerobic environment. The host microbiota will affect ETEC both direct through competition and indirect through generation or competition of available carbon sources in the gut.

Enteric microorganisms such as ETEC exist in two different environments, the environment outside of the human host where they survive and the gastrointestinal tract of the host where they cause infection ( Fig. 1). In the outside environment, ETEC are often present in freshwater bodies, soil, manure, plants and vegetables as a result of faecal contamination. Under such conditions, ETEC has developed mechanism that allows the bacteria to survive. In contrast, when ETEC reaches the gastrointestinal tract, it has developed mechanisms for adaptation to the different conditions bacteria may encounter and has optimized its virulence mechanisms to certain specific conditions in a multifactorial process, regulated by many genes that are expressed at different conditions. Although the contents of the normal gut is usually considered when examining host factors that affect virulence, it is important to remember that the ecology of the small intestine is changing during a diarrhoeal episode when water and electrolytes (sodium, chloride, potassium and bicarbonate) are flushed out in the lumen from infected epithelial cells. These conditions might locally increase or decrease ions, osmolarity and pH and should be taken into account. The changing microbiota and/or temporal shift towards dominance of the infecting strains should also be taken into account since it might generate totally different ecological conditions than in a health gut. It is also important to remember that virulence regulation conditions examined in lab cultures might not apply during infection.

We conclude that a more comprehensive understanding of the pathogenesis of ETEC bacteria in different ecological niches generates important information that can be exploited towards developing methods of controlling infection. Vaccines, oral rehydration solution and antibiotics are commonly used to prevent and treat diarrhoeal diseases, but it is evident that other factors such as diet, food additives and probiotica/microbiota might be able to modify, or even prevent, serious disease. The impact of the bacterial microbiota on ETEC in water and food environments has not yet been addressed at all to our knowledge but could potentially generate means to inhibit transmission pathways. Studies on ecological niches and their influence on survival and virulence of ETEC are therefore important in the continuing fight against diarrhoeal diseases.
